# Hepatitis B virus reactivation and hepatitis in diffuse large B-cell lymphoma patients with resolved hepatitis B receiving rituximab-containing chemotherapy: risk factors and survival

**DOI:** 10.1186/s40880-015-0015-9

**Published:** 2015-05-28

**Authors:** Kai-Lin Chen, Jie Chen, Hui-Lan Rao, Ying Guo, Hui-Qiang Huang, Liang Zhang, Jian-Yong Shao, Tong-Yu Lin, Wen-Qi Jiang, De-Hui Zou, Li-Yang Hu, Michael Lucas Wirian, Qing-Qing Cai

**Affiliations:** Sun Yat-sen University Cancer Center; State Key Laboratory of Oncology in South China; Collaborative Innovation Center of Cancer Medicine, Guangzhou, 510060 Guangdong P. R. China; Department of Medical Oncology, Sun Yat-sen University Cancer Center, Guangzhou, 510060 Guangdong P. R. China; Guangdong Province Key Laboratory of Arrhythmia and Electrophysiology, Radiotherapy Department, Sun Yat-sen Memorial Hospital of Sun Yat-sen University, Guangzhou, 510120 Guangdong P. R. China; Department of Pathology, Sun Yat-sen University Cancer Center, Guangzhou, 510060 Guangdong P. R. China; Clinical Trial Center, Sun Yat-sen University Cancer Center, Guangzhou, 510060 Guangdong P. R. China; Department of Lymphoma and Myeloma, University of Texas MD Anderson Cancer Center, Houston, Texas 77030 USA; Department of Molecular Diagnostics, Sun Yat-sen University Cancer Center, Guangzhou, 510060 Guangdong P. R. China; Lymphoma and Myeloma Center, Institute of Hematology and Blood Diseases Hospital, Tianjin, P. R. China; State Key Lab of Experimental Method of Hematology, Chinese Academy of Medical Sciences and Peking Union of Medical College, Tianjin, 300020 P. R. China

**Keywords:** Diffuse large B-cell lymphoma, Hepatitis, Hepatitis B virus reactivation, Resolved hepatitis B

## Abstract

**Introduction:**

Hepatitis B virus (HBV) reactivation has been reported in B-cell lymphoma patients with resolved hepatitis B (hepatitis B surface antigen [HBsAg]-negative and hepatitis B core antibody [HBcAb]-positive). This study aimed to assess HBV reactivation and hepatitis occurrence in diffuse large B-cell lymphoma (DLBCL) patients with resolved hepatitis B receiving rituximab-containing chemotherapy compared with HBsAg-negative/HBcAb-negative patients to identify risk factors for HBV reactivation and hepatitis occurrence and to analyze whether HBV reactivation and hepatitis affect the survival of DLBCL patients with resolved hepatitis B.

**Methods:**

We reviewed the clinical data of 278 patients with DLBCL treated with rituximab-containing therapy between January 2004 and May 2008 at Sun Yat-sen University Cancer Center, China. Predictive factors for HBV reactivation, hepatitis development, and survival were examined by univariate analysis using the chi-square or Fisher’s exact test and by multivariate analysis using the Cox regression model.

**Results:**

Among the 278 patients, 165 were HBsAg-negative. Among these 165 patients, 6 (10.9%) of 55 HBcAb-positive (resolved HBV infection) patients experienced HBV reactivation compared with none (0%) of 110 HBcAb-negative patients (*P* = 0.001). Patients with resolved hepatitis B had a higher hepatitis occurrence rate than HBsAg-negative/HBcAb-negative patients (21.8% vs. 8.2%, *P* = 0.013). HBcAb positivity and elevated baseline alanine aminotransferase (ALT) levels were independent risk factors for hepatitis. Among the 55 patients with resolved hepatitis B, patients with elevated baseline serum ALT or aspartate aminotransferase (AST) levels were more likely to develop hepatitis than those with normal serum ALT or AST levels (*P* = 0.037, *P* = 0.005, respectively). An elevated baseline AST level was an independent risk factor for hepatitis in these patients. Six patients with HBV reactivation recovered after immediate antiviral therapy, and chemotherapy was continued. HBcAb positivity, HBV reactivation, or hepatitis did not negatively affect the survival of DLBCL patients.

**Conclusions:**

DLBCL patients with resolved hepatitis B may have a higher risk of developing HBV reactivation and hepatitis than HBsAg-negative/HBcAb-negative patients. Close monitoring and prompt antiviral therapy are required in these patients.

## Background

Hepatitis B virus (HBV) infection is a serious global public health problem. It is common in China and other parts of Southeast Asia as well as in the Western Pacific regions [1]. HBV reactivation is a well-recognized complication in cancer patients with chronic HBV infection undergoing immunosuppressive or cytotoxic chemotherapy.

Rituximab is a chimeric mouse/human anti-CD20 monoclonal antibody. Rituximab in combination with cyclophosphamide, hydroxydaunomycin (doxorubicin), vincristine, and prednisone (R-CHOP) is the current standard chemotherapy regimen for diffuse large B-cell lymphoma (DLBCL) [2,3]. Recent evidence has shown that HBV reactivation is associated with the use of rituximab [4]. Without prophylaxis, hepatitis B surface antigen (HBsAg)-positive patients receiving rituximab-containing therapy show a high incidence of HBV reactivation and HBV-related liver failure and death [5-7]. Antiviral prophylaxis is therefore currently recommended for these patients [8]. HBV reactivation can also be observed in lymphoma patients with resolved HBV infection (HBsAg-negative and hepatitis B core antibody [HBcAb]- and/or hepatitis B surface antigen antibody [HBsAb]-positive) during the course of rituximab-containing therapy and may prove to be fatal [4,7,9-13]. The data on the incidence of HBV reactivation and its risk factors as well as the effects of HBV reactivation and hepatitis on the survival of HBsAg-negative DLBCL patients after rituximab-containing therapy are limited in China.

This retrospective study therefore aimed to determine the occurrence rates of HBV reactivation and hepatitis in DLBCL patients with resolved hepatitis B compared with HBsAg-negative/HBcAb-negative patients, to identify risk factors for HBV reactivation and hepatitis in HBsAg-negative patients and patients with resolved hepatitis B, and to analyze whether HBV reactivation or hepatitis could affect the survival of patients with resolved hepatitis B after rituximab-containing therapy.

## Methods

### Patient selection

Between January 2004 and May 2008, patients diagnosed with CD20^+^ DLBCL who were treated with rituximab-containing chemotherapy at Sun Yat-sen University Cancer Center, China were screened for this study. Their HBsAg status was determined before they were administered anticancer therapy.

Patients who were negative for HBsAg underwent HBV serology tests, including those for HBsAb, hepatitis B e antigen (HBeAg), hepatitis B e antibody (HBeAb), and HBcAb. In addition, HBV serology, HBV DNA, and liver function (alanine aminotransferase [ALT], aspartate aminotransferase [AST], and total bilirubin [TB] levels) were tested before each chemotherapy cycle and at least every 3 months during the follow-up period. HBsAg or HBV DNA tests were performed if abnormal liver function was observed or if hepatitis was suspected. Patients enrolled in this study had no evidence of hepatitis A virus (HAV), hepatitis C virus (HCV), hepatitis D virus, hepatitis E virus, or human immunodeficiency virus infection and had adequate available clinical information and follow-up data. The exclusion criteria were the coexistence of another type of lymphoma, associated chronic inflammation, and a previous malignancy or second primary tumor. Hepatitis serology was tested for all patients before they started chemotherapy. HBV DNA was tested using a polymerase chain reaction assay (ABI 7900; Applied Biosystems, Foster City, CA, USA). The lower detection limit for HBV DNA was 100 IU/mL.

This study was approved by the Institutional Review Board of Sun Yat-sen University Cancer Center. Informed consent for the collection of medical information was obtained from all patients at their first visit. All pathologic specimens were reviewed and reclassified according to the World Health Organization (WHO) criteria for pathological diagnosis [14].

### Definitions

Hepatitis and HBV reactivation have been defined previously [15,16]. Hepatitis was defined as a 3-fold or greater increase in serum ALT levels that exceeds the upper limit of normal (ULN) or as an absolute increase in ALT levels to >100 U/L. The ULN of ALT in our hospital is 40 U/L. Hepatitis was attributed to HBV reactivation when there was evidence of HBsAg seroreversion (reappearance of HBsAg), with an increase in HBV DNA levels compared with baseline, in the absence of clinical or laboratory features of acute infection with HAV, HCV, or other systemic infections.

The international prognosis index (IPI) included five factors: age (≤60 years vs. >60 years), lactate dehydrogenase (LDH) value (≤245 U/mL vs. >245 U/mL), Eastern Cooperative Oncology Group (ECOG) performance status (PS) (0–1 vs. >1), Ann Arbor stage (I/II vs. III/IV), and the number of extranodal involvements (0–1 vs. >1). IPI scores were separated based on the number of factors present [17].

### Statistical analysis

Overall survival (OS) was measured from the date of diagnosis to the date of death from any cause or to the date of the last follow-up visit. Variables were examined for associations with hepatitis or HBV reactivation by univariate analysis using χ^2^ or Fisher’s exact test. Multivariate logistic regression analysis was performed to identify predictors of hepatitis or HBV reactivation. Survival curves were drawn by the Kaplan–Meier method and compared using the log-rank test. The prognostic importance of factors was analyzed using the Cox regression model [18]. Multivariate analysis was carried out using a forward stepwise procedure. Factors with a *P* value < 0.2 in the univariate analysis were incorporated into the multivariate analysis. Statistical significance was defined as *P* < 0.05 (two-tailed). Statistical analyses were performed with PASW version 18.0 software (IBM, Armonk, NY, USA).

## Results

### Patient characteristics

**Figure 1:**
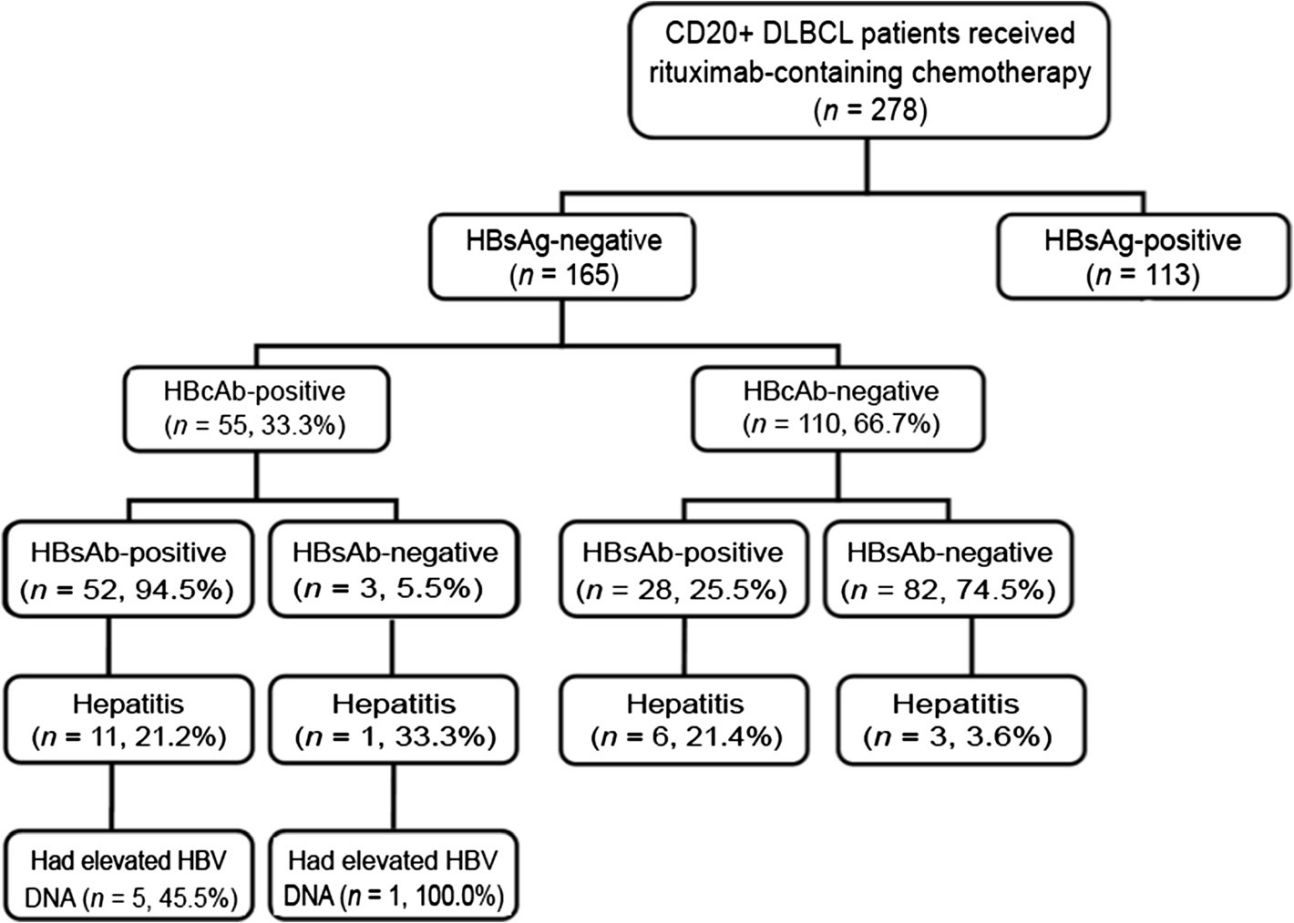
Hepatitis B virus (HBV) status and hepatitis outcome in 278 hepatitis B surface antigen (HBsAg)-negative patients with CD20^+^ diffuse large B-cell lymphoma (DLBCL). HBeAb, hepatitis B e antibody; HBsAb, hepatitis B surface antibody.

Between January 2004 and May 2008, 278 DLBCL patients were treated with rituximab-containing chemotherapy (Figure 1). Among them, 165 were negative for HBsAg. Of these 165 patients, 55 (33.3%) were positive for HBcAb, 80 (48.5%) were positive for HBsAb, and 150 (90.9%) were negative for HBeAb. All patients were negative for HBV DNA before rituximab-containing therapy. The HBsAg-negative patients were predominantly males, with a median age of 54 years (range, 8–83 years). Most of these patients had a favorable PS, no bulky mass, no evidence of B symptoms, no liver, spleen, or bone marrow involvement, and normal liver function. There were no significant differences in the baseline characteristics between HBcAb-positive and -negative patients, except for the positive rate of HBsAb (94.5% vs. 25.5%, *P* < 0.001). The median age was 56 years (range, 8 to 83 years) for HBcAb-negative patients and 58 years (range, 18 to 79 years) for HBcAb-positive patients (*P =* 0.798). The median number of rituximab-containing chemotherapy cycles was 4 (range, 1 to 8) for HBcAb-negative patients and 4 (range, 1 to 7) for HBcAb-positive patients (*P =* 0.343). The detailed clinical characteristics are listed in Table 1.

**Table 1:** **Baseline clinical characteristics according to HBcAb status in HBsAg-negative DLBCL patients**

**Characteristic**	**HBcAb-negative [cases (%)]**	**HBcAb-positive [cases (%)]**	***P*** **value**
Total	110	55	
Sex			0.434
Male	67 (60.9)	30 (54.5)	
Female	43 (39.1)	25 (45.5)	
ECOG PS			0.525
0–1	96 (87.3)	46 (83.6)	
≥2	14 (12.7)	9 (16.4)	
Bulky mass			0.619
Yes	28 (25.5)	16 (29.1)	
No	82 (74.5)	39 (70.9)	
B symptoms			0.448
−	80 (72.7)	43 (78.2)	
+	30 (27.3)	12 (21.8)	
Ann Arbor stage^†^			0.698
I–II	52 (47.3)	28 (50.9)	
III-IV	57 (51.8)	27 (49.1)	
Liver involvement			0.720
−	104 (94.5)	53 (96.4)	
+	6 (5.5)	2 (3.6)	
IPI score^†^			0.851
0–1	48 (43.6)	21 (38.2)	
2	28 (25.4)	17 (30.9)	
3	23 (20.9)	11 (20.0)	
4-5	10 (9.1)	6 (10.9)	
ALT			0.874
≤40 U/L	95 (86.4)	47 (85.5)	
>40 U/L	15 (13.6)	8 (14.5)	
AST			0.863
≤45 U/L	97 (88.2)	49 (89.1)	
>45 U/L	13 (11.8)	6 (10.9)	
TB			0.852
≤20.5 μmol/L	99 (90.0)	50 (90.9)	
>20.5 μmol/L	11 (10.0)	5 (9.1)	
LDH			0.910
≤245 U/mL	67 (60.9)	33 (60.0)	
>245 U/mL	43 (39.1)	22 (40.0)	
HBsAb			<0.001
–	82 (74.5)	3 (5.5)	
+	28 (25.5)	52 (94.5)	
HBeAb			0.251
–	102 (92.7)	48 (87.3)	
+	8 (7.3)	7 (12.7)	

HBcAb, hepatitis B core antibody; HBsAg, hepatitis B surface antigen; DLBCL, diffuse large B-cell lymphoma; ECOG PS, Eastern Cooperative Oncology Group performance status; IPI, international prognostic index; ALT, alanine aminotransferase; AST, aspartate aminotransferase; TB, total bilirubin; LDH, lactate dehydrogenase; HBsAb, hepatitis B surface antibody; HBeAb, hepatitis B e antibody. ^†^The information of Ann Arbor stage and IPI were available for 164 patients.

### HBV reactivation rate and its risk factors in DLBCL patients with resolved hepatitis B after rituximab-containing chemotherapy

Overall, 6 patients developed HBV reactivation after rituximab-containing chemotherapy, and they all had resolved HBV infection. Among the 110 HBsAg-negative/HBcAb-negative patients, 9 developed hepatitis, but none were associated with HBV reactivation (Figure 1). Univariate analysis showed that HBsAg-negative/HBcAb-positive patients had a greater likelihood of developing HBV reactivation than HBsAg-negative/HBcAb-negative patients (10.9% vs. 0%, *P* = 0.001). There were no significant difference between patients with and without HBV reactivation in terms of age, sex, ECOG PS, Ann Arbor disease stage, bulky mass presence, B symptoms, IPI score, liver, spleen, extranodal site, or bone marrow involvement, and ALT, AST, TB, or LDH levels (all *P* > 0.05) (Table 2). Among the 55 patients with resolved hepatitis B, there were no significant differences between patients with and without HBV reactivation in terms of the above mentioned variables (all *P* > 0.05).

**Table 2:** **Univariate analysis of potential risk factors for HBV reactivation in HBsAg-negative DLBCL patients**

**Factor**	**Without HBV reactivation [cases (%)]**	**With HBV reactivation [cases (%)]**	***P*** **value**
Total	159	6	
HBcAb			0.001
–	110 (69.2)	0 (0)	
+	49 (30.8)	6 (100.0)	
Age (years)			0.237
≤60	65 (40.9)	4 (66.7)	
>60	94 (59.1)	2 (33.3)	
Sex			1.000
Female	66 (41.5)	2 (33.3)	
Male	93 (58.5)	4 (66.7)	
ECOG PS			1.000
0–1	137 (86.2)	5 (83.3)	
≥2	22 (13.8)	1 (16.7)	
Bulky mass			0.343
Yes	115 (72.3)	6 (100.0)	
No	44 (27.7)	0 (0)	
B symptoms			1.000
–	118 (74.2)	5 (83.3)	
+	41 (25.8)	1 (16.7)	
Ann Arbor stage^†^			0.211
I–II	79 (49.7)	1 (16.7)	
III-IV	79 (49.7)	5 (83.3)	
Liver involvement			1.000
–	151 (95.0)	6 (100.0)	
+	8 (5.0)	0 (0)	
Spleen involvement			0.441
–	145 (91.2)	5 (83.3)	
+	14 (8.8)	1 (16.7)	
Involved extranodal sites			0.627
<2	122 (76.7)	4 (66.7)	
≥2	37 (23.3)	2 (33.3)	
Bone marrow involvement			1.000
–	148 (93.1)	6 (100.0)	
+	11 (6.9)	0 (0)	
IPI score^†^			0.402
0–1	68 (42.8)	1 (16.7)	
2–5	90 (56.6)	5 (83.3)	
HBeAb			0.094
–	146 (91.8)	4 (66.7)	
+	13 (8.2)	2 (33.3)	
ALT			0.597
≤40 U/L	136 (85.5)	6 (100.0)	
>40 U/L	23 (14.5)	0 (0)	
AST			0.526
≤45 U/L	141 (88.7)	5 (83.3)	
>45 U/L	18 (11.3)	1 (16.7)	
TB			0.463
≤20.5 μmol/L	144 (90.6)	5 (83.3)	
>20.5 μmol/L	15 (9.4)	1 (16.7)	
LDH			0.681
≤245 U/mL	97 (61.0)	3 (50.0)	
>245 U/mL	62 (39.0)	3 (50.0)	

HBV, hepatitis B virus. Other footnotes as in Table 1.

### Hepatitis rate and its risk factors in DLBCL patients with resolved hepatitis B after rituximab-containing chemotherapy

Hepatitis was observed in 21 (12.7%) of the 165 HBsAg-negative CD20^+^ DLBCL patients, 6 of whom had HBV-related hepatitis. Other possible causes of hepatitis were chemotherapy (*n* = 7), tumor progression (*n* = 4), heart failure (*n* = 1), infection (*n* = 2), and septic shock (*n* = 1). For the 3 HBsAg-, HBcAb-, and HBsAb-negative patients, the possible causes of hepatitis were chemotherapy (*n* = 2) and tumor progression (*n* = 1). Univariate analysis identified a higher incidence of hepatitis in the HBcAb-positive group compared with the HBcAb-negative group (21.8% vs. 8.2%, *P* = 0.013). Moreover, patients with elevated serum ALT levels at baseline were more likely to develop hepatitis than those with normal serum ALT levels (30.4% vs. 9.9%, *P* = 0.013). The risk of hepatitis was also higher in patients with elevated serum AST levels at baseline than in those with normal serum AST levels (31.6% vs. 10.3%, *P* = 0.019). The univariate analysis results of potential prognostic factors associated with hepatitis are shown in Table 3. Multivariate logistic regression analysis revealed that HBcAb positivity and elevated serum ALT levels at baseline were independently associated with the development of hepatitis in HBsAg-negative DLBCL patients (both *P* < 0.05) (Table 4).

**Table 3:** **Univariate analysis of potential prognostic factors associated with hepatitis in HBsAg-negative DLBCL patients**

**Factor**	**Without hepatitis [cases (%)]**	**With hepatitis [cases (%)]**	***P*** **value**
Total	144	21	
HBcAb			0.013
–	101 (70.1)	9 (42.9)	
+	43 (29.9)	12 (57.1)	
HBeAb			1.000
–	131 (91.0)	19 (90.5)	
+	13 (9.0)	2 (9.5)	
Age (years)			0.399
≤60	62 (43.1)	7 (33.3)	
>60	82 (56.9)	14 (66.7)	
Sex			0.756
Female	60 (41.7)	8 (38.1)	
Male	84 (58.3)	13 (61.9)	
ECOG PS			0.500
0–1	125 (86.8)	17 (81.0)	
≥2	19 (13.2)	4 (19.0)	
B symptoms			0.726
−	108 (75.0)	15 (71.4)	
+	36 (25.0)	6 (28.6)	
Ann Arbor stage^†^			0.129
I–II	73 (50.7)	7 (33.3)	
III-IV	70 (48.6)	14 (66.7)	
Liver involvement			0.598
−	136 (94.4)	21 (100)	
+	8 (5.6)	0 (0)	
Spleen involvement			1.000
−	131 (91.0)	19 (90.5)	
+	13 (9.0)	2 (9.5)	
Involved extranodal sites			0.586
<2	111 (77.1)	15 (71.4)	
≥2	33 (22.9)	6 (28.6)	
Bone marrow involvement			1.000
−	134 (93.1)	20 (95.2)	
+	10 (6.9)	1 (4.8)	
IPI score^†^			0.180
0–1	63 (43.8)	6 (28.6)	
2–5	80 (55.8)	15 (71.4)	
ALT			0.013
≤40 U/L	128 (88.9)	14 (66.7)	
>40 U/L	16 (11.1)	7 (33.3)	
AST			0.019
≤45 U/L	131 (91.0)	15 (71.4)	
>45 U/L	13 (9.0)	6 (28.6)	
TB			0.696
≤20.5 μmol/L	129 (89.6)	20 (95.2)	
>20.5 μmol/L	15 (10.4)	1 (4.8)	
LDH			0.728
≤245 U/mL	88 (61.1)	12 (57.1)	
>245 U/mL	56 (38.9)	9 (42.9)	

Footnotes as in Table 1.

**Table 4:** **Multivariate analysis of potential risk factors associated with hepatitis in HBsAg-negative DLBCL patients**

**Variate**	**OR**	**95% CI**	***P*** **value**
HBcAb (+)	3.27	1.25-8.59	0.016
ALT (>40 U/L)	4.22	1.43-12.49	0.009
AST(>45 U/L)	NA	NA	0.129

OR, odds ratio; CI, confidence interval; NA, not applicable. Other abbreviations as in Table 1.

For the 55 patients with resolved hepatitis B, 22 (40%) had elevated serum ALT levels during the chemotherapy and follow-up period, and 12 (21.8%) experienced hepatitis. The possible causes of hepatitis were HBV-related disease (*n* = 6), chemotherapy (n = 1), tumor progression (*n* = 2), heart failure (*n* = 1), and infection (*n* = 2). Patients with elevated serum ALT or AST levels at baseline were more likely to develop hepatitis than those with normal serum ALT or AST levels (*P* = 0.037 and *P* = 0.005, respectively). The univariate analysis results of potential prognostic factors associated with hepatitis in patients with resolved hepatitis B are shown in Table 5. Multivariate logistic regression analysis revealed that elevated serum AST levels at baseline were independently associated with the development of hepatitis in DLBCL patients with resolved hepatitis B (odds ratio [OR] = 10.25, 95% confidence interval [CI] = 1.60–21.73, *P* = 0.014).

**Table 5:** **Univariate analysis of potential prognostic factors associated with hepatitis in DLBCL patients with resolved hepatitis B**

**Factor**	**Without hepatitis [cases (%)]**	**With hepatitis [cases (%)]**	***P*** **value**
Total	43	12	
HBeAb			0.639
–	38 (88.4)	10 (83.3)	
+	5 (11.6)	2 (16.7)	
Age (years)			0.831
≤60	23 (53.5)	6 (50.0)	
>60	20 (46.5)	6 (50.0)	
Sex			0.340
Female	21 (48.8)	4 (33.3)	
Male	22 (51.2)	8(66.7)	
ECOG PS			0.974
0–1	36 (83.7)	10 (83.3)	
≥2	7 (16.3)	2 (16.7)	
B symptoms			0.763
−	34 (79.1)	9 (75.0)	
+	9 (20.9)	3 (25.0)	
Ann Arbor stage			0.168
I–II	24 (55.8)	4 (33.3)	
III-IV	19 (44.2)	8 (66.7)	
Liver involvement			0.447
−	41 (95.3)	12 (100)	
+	2 (4.7)	0 (0)	
Spleen involvement			0.873
−	40 (93.0)	11 (91.7)	
+	3 (7.0)	1 (8.3)	
Involved extranodal sites			0.275
<2	35 (81.4)	8 (66.7)	
≥2	8 (18.6)	4 (33.3)	
Bone marrow involvement			0.594
−	42 (97.7)	12 (100.0)	
+	1 (2.3)	0 (0.0)	
IPI score			0.288
0–1	18 (41.9)	3 (25.0)	
2–5	25 (58.1)	9 (75.0)	
ALT			0.037
≤40 U/L	39 (90.7)	8 (66.7)	
>40 U/L	4 (9.3)	4 (33.3)	
AST			0.005
≤45 U/L	41 (95.3)	8 (66.7)	
>45 U/L	2 (4.7)	4 (33.3)	
TB			0.918
≤20.5 μmol/L	39 (90.7)	11 (91.7)	
>20.5 μmol/L	4 (9.3)	1 (8.3)	
LDH			0.894
≤245 U/mL	26 (60.5)	7 (58.3)	
>245 U/mL	17 (39.5)	5 (41.7)	

Abbreviations as in Table 1.

### Details of 6 patients who developed HBV reactivation

Details of the patients who developed HBV reactivation are shown in Table 6. Most of these patients were males, were older (>60 years), and had bulky masses, advanced Ann Arbor stage, and HBsAb positivity at baseline. No patient showed detectable serum HBV DNA levels at baseline. Most of the patients had normal liver function at baseline, except for Patients 1 and 2. None of the patients received antiviral prophylaxis.

**Table 6:** **Details and outcomes of 6 HBsAg-negative/HBcAb-positive DLBCL patients who developed HBV reactivation after rituximab-containing chemotherapy**

**Patient no.**	**Age (years)**	**Sex**	**Baseline**						**R-CHOP cycles**	**Interval between last cycle and HBV reactivation (days)**	**At diagnosis of HBV reactivation**				**Outcome**
			**IPI**	**Stage**	**HBeAb**	**ALT (U/L)**	**AST (U/L)**	**TB (μmol/L)**			**Peak HBV DNA (IU/mL)**	**Peak ALT (U/L)**	**Peak AST (U/L)**	**Peak TB (μmol/L)**	
1	65	Male	4	3	+	18.0	68.2	8.9	5	42	1.72 × 10^7^	333.1	285.8	69.0	Alive and well
2	21	Male	3	3	-	22.0	27.0	36.6	2	24	8.60 × 10^3^	107.6	60.2	47.1	Died of lymphoma
3	69	Female	2	3	-	21.0	25.0	9.4	6	23	1.80 × 10^5^	230.8	120.1	11.0	Alive and well
4	70	Male	3	3	-	10.0	29.0	11.1	7	98	4.76 × 10^6^	239.0	174.0	8.1	Died of lymphoma
5	26	Female	2	3	+	10.0	15.0	6.4	2	27	9.28 × 10^6^	106.0	89.0	7.8	Alive and well
6	68	Male	1	1	-	25.0	35.0	7.0	6	29	4.47 × 10^4^	219.2	204.5	11.3	Alive and well

R-CHOP, cyclophosphamide, hydroxydaunomycin (doxorubicin), vincristine, and prednisone regimen. Other abbreviations as in Tables 1 and 2.

HBV reactivation was more likely to occur after an average of 5 R-CHOP cycles (range, 2–7 cycles) at a median interval of 27 days (range, 24–98 days). Antiviral drug administration was immediately started after HBV DNA detection. Three patients developed severe hepatitis. Hepatoprotective drugs were used when necessary, and HBV reactivation was managed with lamivudine (100 mg/day). Rituximab-containing chemotherapy was continued after serum HBV DNA became undetectable and liver function had improved.

### Survival of DLBCL patients with resolved hepatitis B after rituximab-containing chemotherapy

**Figure 2:**
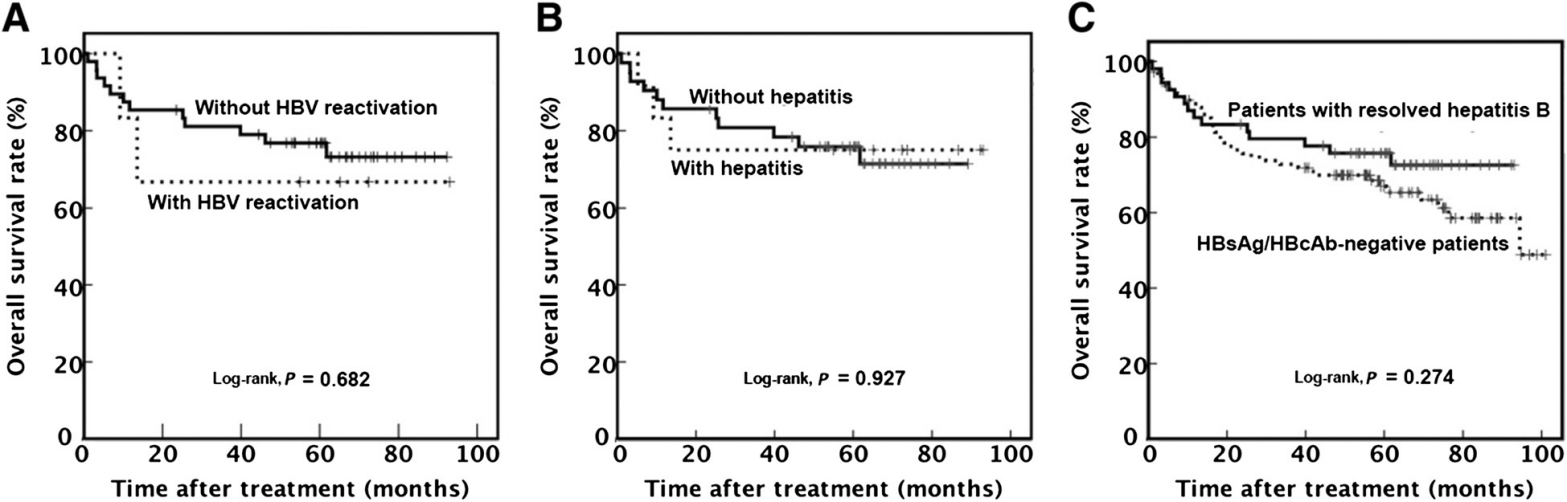
Overall survival (OS) curves for DLBCL patients. **A**, the trends of OS curves for patients with or without HBV reactivation during rituximab-containing chemotherapy were similar. **B**, the trends of OS curves for patients with or without hepatitis were similar. **C**, the trends of OS curves for patients with resolved hepatitis B and HBsAg-negative/HBcAb-negative patients were also similar. Abbreviations as in Figure 1.

Among patients with resolved hepatitis B, the median follow-up duration was 61 (1–93) months, the median 1-year OS rate was 85.2% (95% CI, 75.8%–100.0%), the 3-year OS rate was 79.5% (95% CI, 68.7%–90.2%), and the 5-year OS rate was 75.7% (95% CI, 64.1%–87.2%). Among these patients, no significant difference was observed in OS between patients with and without HBV reactivation (66.7% [95% CI, 29.0%–100%] vs. 73.2% [95% CI, 59.9%–86.5%], *P* = 0.682) (Figure 2A) or between patients with and without hepatitis (75.0% [95% CI, 50.5%–99.5%] vs. 71.4% [95% CI, 56.5%–86.3%], *P* = 0.927) (Figure 2B). OS was similar in patients with resolved hepatitis B and HBsAg-negative/HBcAb-negative patients (75.7% [95% CI, 64.1%–87.2%] vs. 68.5% [95% CI, 57.8%–76.2%], *P* = 0.274) (Figure 2C). Among HBsAg-negative patients, no significant difference was observed in OS between patients with and without HBV reactivation (66.7% vs. 70.2%, *P* = 0.908) or between patients with and without hepatitis (75.2% vs. 71.3%, *P* = 0.512).

## Discussion

This study demonstrated that CD20^+^ DLBCL patients with resolved HBV infection had a significantly higher risk of HBV reactivation and hepatitis compared with HBsAg-negative/HBcAb-negative patients after rituximab-containing chemotherapy. Baseline HBcAb positivity and elevated baseline serum ALT levels were independent risk factors for hepatitis in HBsAg-negative patients. An elevated baseline AST level was an independent risk factor for hepatitis in patients with resolved hepatitis B. HBV reactivation could be managed with prompt antiviral therapy followed by the continuation of chemotherapy. HBcAb positivity, HBV reactivation, or hepatitis did not negatively affect patient survival.

Several studies have consistently demonstrated that the incidence of HBV infection is higher in patients with B-cell lymphoma than in the general population [19-22]. Chronic HBV infection was considered to be associated with lymphomagenesis [23], especially for B-cell lymphoma [24]. In this study, 33.3% (55/165) of patients had resolved HBV infection, which was consistent with the 20%–40% incidence reported in endemic areas [25]. The incidence of HBV reactivation was 3.6% (6/165) in HBsAg-negative patients in this study, but was reported as high as 80% (12/15) in HBsAg-positive lymphoma patients receiving rituximab-containing chemotherapy without prophylaxis [5].

Compared with HBsAg-negative/HBcAb-negative patients, HBsAg-negative/HBcAb-positive patients had a greater likelihood of developing HBV reactivation (10.9% vs. 0%, *P* = 0.001). However, Hui *et al*. [9] found no significant difference between the two groups in a study of non-Hodgkin’s lymphoma and Hodgkin lymphoma patients, of whom only 20.1% (49/244) received R-CHOP therapy. The incidence of HBV reactivation varied from 2.3% to 23.8% of patients with resolved HBV infection [4,10-13]. The reasons for this discrepancy remain to be elucidated. However, treatment intensity, patient characteristics, and geographic HBV differences may be responsible. Among the patients with resolved hepatitis B, no significant risk factors for HBV reactivation were found. Prospective studies with larger sample sizes are required to investigate the risk factors for resolved hepatitis B.

Hepatitis can be caused by a range of factors, including tumor progression, drug-related factors, heart failure, and severe sepsis. In this study, hepatitis was caused by HBV reactivation in 28.5% (6/21) of patients, and HBV reactivation was accompanied by hepatitis in all of the 6 patients, given that HBV reactivation often causes a disease flare that leads to liver dysfunction. However, HBV reactivation can also be transient and clinically silent if the viral load is low [26]; thus, HBV reactivation can also occur in the absence of hepatitis [27,28].

Resolved hepatitis B involves interaction between the virus and the immune system. A variety of mechanisms may be involved. Low-level HBV virus replication is the main cause of resolved HBV infection. Other causes include HBV gene mutation, HBV integration into the host chromosome, HBV infection of peripheral mononuclear cells, host immune response abnormalities, and interference by other viral infection. Persistent HBV infection can promote liver disease, thereby leading to hepatitis and cirrhosis [29].

ALT is primarily localized in the liver, with lower enzymatic activities found in skeletal muscle and heart tissues. AST is localized in heart, brain, skeletal muscle, and liver tissues. Damaged hepatocytes release their contents, including ALT and AST, into the extracellular space [30]. Serum ALT or AST levels are generally considered sensitive indicators of liver cell injury. However, in addition to HBV infection, elevated baseline serum ALT levels can be caused by various other factors, including tumor progression, fatty liver, and diabetes [31]. In contrast to our results, Yeo *et al*. [4] found that all the patients who developed HBV reactivation had normal baseline serum ALT levels and that the incidence of hepatitis was similar in HBsAg-negative/HBcAb-negative patients and in patients with resolved HBV infection. However, 53.7% (43/80) of patients in that study were treated without rituximab. Further studies are therefore needed to clarify the relationship between elevated baseline serum ALT levels and the hepatitis risk, as well as the possible mechanisms involved.

Among the 55 HBcAb-positive patients in our study, 8 (14.5%) had elevated baseline serum ALT levels. During the chemotherapy and follow-up period, 22 (40%) of the 55 patients had elevated serum ALT levels. Data on the proportion of HBcAb-positive patients with elevated serum ALT levels were limited. In a study by Yeo *et al*. [4], the incidence of elevated serum ALT levels in HBcAb-positive patients was 19.6% during chemotherapy. The reasons for these differences remain unclear. However, differences in patient characteristics and treatment intensities may be responsible. Therefore, there is no obvious association between HBcAb-positive patients and ALT levels, and further studies are warranted.

Several studies demonstrated that rituximab obviously improved outcomes in patients with B-cell lymphoma [2,3]. However, rituximab has been reported to increase the HBV reactivation rate in patients with chronic HBV infection or resolved HBV infection. Although HBV infection control is mediated mainly by HBV-specific cytotoxic T lymphocytes, B lymphocytes are still required for antigen presentation. Rituximab is a chimeric murine/human anti-CD20 monoclonal antibody that alters the activity of T lymphocytes and destroys B lymphocytes, resulting in the failure of antigen presentation and the subsequent expansion of HBV infection in hepatocytes [32,33]. HBV-related hepatitis during rituximab-containing chemotherapy can be severe and fatal [34-36].

According to previous data and our experience, HBV-related hepatitis often develops after 5 or 6 cycles of chemotherapy at an interval of 1 to 13 months after chemotherapy. Antiviral drugs successfully controlled HBV reactivation in most patients, although 2 elderly patients (ages 77 and 84 years) developed fatal HBV-related disease [4,13,37]. In this study, it was reasonable for such patients to undergo close monitoring of HBV serology, HBV DNA, and liver function before each chemotherapy cycle and at least every 3 months during the follow-up period; this is consistent with the monitoring frequency of 1–3 months recommended by the latest European Association for the Study of the Liver (EASL) Clinical Practice Guidelines [8] and the consensus on the management of lymphoma with HBV infection in China [38]. In addition, in this study, HBsAg and HBV DNA testing was performed in the event of abnormal liver function or suspected hepatitis. Lamivudine successfully controlled HBV reactivation in all 6 affected patients. Prophylactic agents thus may not be recommended for HBsAg-negative/HBcAb-positive patients if close monitoring of HBV DNA is guaranteed.

However, regular monitoring may not be suitable for all patients. Some experts recommend prophylaxis with antiviral drugs in all HBsAg-negative/HBcAb-positive patients who receive rituximab-containing regimens for hematologic malignancies with a high risk of HBV reactivation and/or if close monitoring of HBV DNA is not guaranteed [8]. Lamivudine is widely used for prophylaxis; however, its efficacy is hampered by the development of viral mutations that result in drug resistance [39]. The prophylactic entecavir was found to be effective and associated with minimal resistance in lymphoma patients with resolved hepatitis B [40], but its long-term use is expensive. More large-scale studies or meta-analyses are required to identify host and viral factors that can help to predict the occurrence of HBV-related hepatitis and thus allow the design of individualized strategies for preventing HBV-related hepatitis. Different approaches based on cost-effectiveness, particularly in HBV-endemic areas, could be used for patients with different risk levels, and antiviral prophylaxis should be continued indefinitely.

In HBsAg-positive patients with non-Hodgkin’s lymphoma, the HBV reactivation-associated mortality was 30%–50% without antiviral prophylaxis [5]. The HBV-related morbidity and overall mortality remained high in lymphoma patients treated with antiviral prophylaxis [41]. In this study, OS was similar in HBsAg-negative/HBcAb-positive patients and HBsAg-negative/HBcAb-negative patients after rituximab-containing chemotherapy. Moreover, no significant difference was observed in OS between patients with and without HBV reactivation or between patients with and without hepatitis. Fukushima *et al*. [11] found similar survival rates in HBcAb-positive or HBcAb-negative patients, although their study included other types of lymphoma in addition to DLBCL, and 26.4% (19/72) of patients had not received rituximab-containing therapy. In addition, DLBCL is a heterogeneous disorder with varied clinical outcomes. Hsu *et al*. [28] also demonstrated that resolved HBV infection with HBV reactivation was associated with low OS and progression-free survival rates, although the differences were not significant. Further studies are needed to determine the impact of HBV reactivation on the clinical outcomes of lymphoma patients.

Our study had several key points. First, all patients had newly diagnosed DLBCL and received rituximab-containing therapy. Second, we compared the incidence of HBV reactivation or hepatitis between HBcAb-positive and HBcAb-negative patients and identified risk factors for the occurrence of HBV reactivation and hepatitis in HBsAg-negative patients and patients with resolved hepatitis B. Third, we analyzed the effects of HBcAb positivity, HBV reactivation, and hepatitis on survival, which are currently unknown. Furthermore, South China is a highly endemic HBV area, and this is the first to confirm these findings in the southern Chinese population in the largest cancer center in South China.

However, this study was limited by its retrospective nature. First, the assignment of rituximab-containing chemotherapy was not randomized in HBcAb-positive or HBcAb-negative patients but was instead based on the consideration of individual patients, leading to inevitable bias in terms of which patients received rituximab. Second, this study was limited to the analysis of patients from a single institute. All of the patients were Chinese, and our findings therefore must be confirmed in patients from other parts of Asia and in other ethnic groups.

## Conclusions

In conclusion, this study clearly indicates that patients with resolved hepatitis B are at a higher risk of developing HBV reactivation and hepatitis after rituximab-containing chemotherapy compared with HBsAg-negative/HBcAb-negative patients. Close monitoring of HBV DNA levels and liver function and prompt antiviral therapy are required in these patients. Prospective studies including more patients are required to confirm our findings and to determine the most effective monitoring and therapeutic strategies.

## References

[1] Lee WM (1997). Hepatitis B virus infection. N Engl J Med.

[2] Coiffier B, Lepage E, Briere J, Herbrecht R, Tilly H, Bouabdallah R (2002). CHOP chemotherapy plus rituximab compared with CHOP alone in elderly patients with diffuse large-B-cell lymphoma. N Engl J Med.

[3] Pfreundschuh M, Trumper L, Osterborg A, Pettengell R, Trneny M, Imrie K (2006). CHOP-like chemotherapy plus rituximab versus CHOP-like chemotherapy alone in young patients with good-prognosis diffuse large-B-cell lymphoma: a randomised controlled trial by MabThera International Trial (MInT) Group. Lancet Oncol.

[4] Yeo W, Chan TC, Leung NW, Lam WY, Mo FK, Chu MT (2009). Hepatitis B virus reactivation in lymphoma patients with prior resolved hepatitis B undergoing anticancer therapy with or without rituximab. J Clin Oncol.

[5] Shih LN, Sheu JC, Wang JT, Huang GT, Yang PM, Lee HS (1990). Serum hepatitis B virus DNA in healthy HBsAg-negative Chinese adults evaluated by polymerase chain reaction. J Med Virol.

[6] Evens AM, Jovanovic BD, Su YC, Raisch DW, Ganger D, Belknap SM (2011). Rituximab-associated hepatitis B virus (HBV) reactivation in lymphoproliferative diseases: meta-analysis and examination of FDA safety reports. Ann Oncol.

[7] Pei SN, Chen CH, Lee CM, Wang MC, Ma MC, Hu TH (2010). Reactivation of hepatitis B virus following rituximab-based regimens: a serious complication in both HBsAg-positive and HBsAg-negative patients. Ann Hematol.

[8] European Association For The Study Of The Liver (2012). EASL clinical practice guidelines: management of chronic hepatitis B virus infection. J Hepatol.

[9] Hui CK, Cheung WW, Zhang HY, Au WY, Yueng YH, Leung AY (2006). Kinetics and risk of de novo hepatitis B infection in HBsAg-negative patients undergoing cytotoxic chemotherapy. Gastroenterology.

[10] Koo YX, Tay M, Teh YE, Teng D, Tan DS, Tan IB (2011). Risk of hepatitis B virus (HBV) reactivation in hepatitis B surface antigen negative/hepatitis B core antibody positive patients receiving rituximab-containing combination chemotherapy without routine antiviral prophylaxis. Ann Hematol.

[11] Fukushima N, Mizuta T, Tanaka M, Yokoo M, Ide M, Hisatomi T (2009). Retrospective and prospective studies of hepatitis B virus reactivation in malignant lymphoma with occult HBV carrier. Ann Oncol.

[12] Ji D, Cao J, Hong X, Li J, Wang J, Chen F (2010). Low incidence of hepatitis B virus reactivation during chemotherapy among diffuse large B-cell lymphoma patients who are HBsAg-negative/HBcAb-positive: a multicenter retrospective study. Eur J Haematol.

[13] Matsue K, Kimura S, Takanashi Y, Iwama K, Fujiwara H, Yamakura M (2010). Reactivation of hepatitis B virus after rituximab-containing treatment in patients with CD20-positive B-cell lymphoma. Cancer.

[14] Swerdlow SHCE, Harris NL, Jaffe ES, Stein H, Thiele J (2008). World health organization classification of tumours of the haematopoietic and lymphoid tissues.

[15] Yeo W, Chan PK, Ho WM, Zee B, Lam KC, Lei KI (2004). Lamivudine for the prevention of hepatitis B virus reactivation in hepatitis B s-antigen seropositive cancer patients undergoing cytotoxic chemotherapy. J Clin Oncol.

[16] Lok AS, Liang RH, Chiu EK, Wong KL, Chan TK, Todd D (1991). Reactivation of hepatitis B virus replication in patients receiving cytotoxic therapy. Report of a prospective study. Gastroenterology.

[17] Ziepert M, Hasenclever D, Kuhnt E, Glass B, Schmitz N, Pfreundschuh M (2010). Standard international prognostic index remains a valid predictor of outcome for patients with aggressive CD20+ B-cell lymphoma in the rituximab era. J Clin Oncol.

[18] Cox DR (1972). Regression models and life tables. J R Stat Soc B.

[19] Marcucci F, Mele A, Spada E, Candido A, Bianco E, Pulsoni A (2006). High prevalence of hepatitis B virus infection in B-cell non-Hodgkin’s lymphoma. Haematologica.

[20] Wang F, Xu RH, Han B, Shi YX, Luo HY, Jiang WQ (2007). High incidence of hepatitis B virus infection in B-cell subtype non-Hodgkin lymphoma compared with other cancers. Cancer.

[21] Kim JH, Bang YJ, Park BJ, Yoo T, Kim CW, Kim TY (2002). Hepatitis B virus infection and B-cell non-Hodgkin’s lymphoma in a hepatitis B endemic area: a case–control study. Jpn J Cancer Res.

[22] Qin XT, Lu Y, Chen XQ, Xu HP, Fan HJ (2007). Correlation of hepatitis B virus infection to non-Hodgkin’s lymphoma. Chin J Cancer.

[23] Engels EA, Cho ER, Jee SH (2010). Hepatitis B virus infection and risk of non-Hodgkin lymphoma in South Korea: a cohort study. Lancet Oncol.

[24] Wang F, Yuan S, Teng KY, Garcia-Prieto C, Luo HY, Zeng MS (2012). High hepatitis B virus infection in B-cell lymphoma tissue and its potential clinical relevance. Eur J Cancer Prev.

[25] Marzano A, Angelucci E, Andreone P, Brunetto M, Bruno R, Burra P (2007). Prophylaxis and treatment of hepatitis B in immunocompromised patients. Dig Liver Dis.

[26] Hoofnagle JH (2009). Reactivation of hepatitis B. Hepatology.

[27] Li HR, Huang JJ, Guo HQ, Zhang X, Xie Y, Zhu HL (2011). Comparison of entecavir and lamivudine in preventing hepatitis B reactivation in lymphoma patients during chemotherapy. J Viral Hepat.

[28] Hsu C, Tsou HH, Lin SJ, Wang MC, Yao M, Hwang WL (2014). Chemotherapy-induced hepatitis B reactivation in lymphoma patients with resolved HBV infection: a prospective study. Hepatology.

[29] Chemin I, Jeantet D, Kay A, Trepo C (2001). Role of silent hepatitis B virus in chronic hepatitis B surface antigen(−) liver disease. Antiviral Res.

[30] Ozer J, Ratner M, Shaw M, Bailey W, Schomaker S (2008). The current state of serum biomarkers of hepatotoxicity. Toxicology.

[31] Clark JM, Brancati FL, Diehl AM (2003). The prevalence and etiology of elevated aminotransferase levels in the United States. Am J Gastroenterol.

[32] Stasi R, Del Poeta G, Stipa E, Evangelista ML, Trawinska MM, Cooper N (2007). Response to B-cell depleting therapy with rituximab reverts the abnormalities of T-cell subsets in patients with idiopathic thrombocytopenic purpura. Blood.

[33] Dai MS, Chao TY, Kao WY, Shyu RY, Liu TM (2004). Delayed hepatitis B virus reactivation after cessation of preemptive lamivudine in lymphoma patients treated with rituximab plus CHOP. Ann Hematol.

[34] Tsutsumi Y, Kanamori H, Mori A, Tanaka J, Asaka M, Imamura M (2005). Reactivation of hepatitis B virus with rituximab. Expert Opin Drug Saf.

[35] Sarrecchia C, Cappelli A, Aiello P (2005). HBV reactivation with fatal fulminating hepatitis during rituximab treatment in a subject negative for HBsAg and positive for HBsAb and HBcAb. J Infect Chemother.

[36] Li YH, He YF, Wang FH, Lin XB, Xia ZJ, Sun XF (2005). Clinical analysis of liver damage of 116 malignant lymphoma patients with chronic HBV infection after cytotoxic chemotherapy. Chin J Cancer.

[37] Koo YX, Tan DS, Tan IB, Tao M, Chow WC, Lim ST (2010). Hepatitis B virus reactivation and role of antiviral prophylaxis in lymphoma patients with past hepatitis B virus infection who are receiving chemoimmunotherapy. Cancer.

[38] Chinese Society of Hematology, CMA, Committee of Malignant Lymphoma, Chinese Anti-cancer Association, Chinese Society of Hepatology, CMA (2013). Consensus on the management of lymphoma with HBV infection. Zhonghua Xue Ye Xue Za Zhi.

[39] Lok AS, Lai CL, Leung N, Yao GB, Cui ZY, Schiff ER (2003). Long-term safety of lamivudine treatment in patients with chronic hepatitis B. Gastroenterology.

[40] Huang YH, Hsiao LT, Hong YC, Chiou TJ, Yu YB, Gau JP (2013). Randomized controlled trial of entecavir prophylaxis for rituximab-associated hepatitis B virus reactivation in patients with lymphoma and resolved hepatitis B. J Clin Oncol.

[41] Kumagai K, Takagi T, Nakamura S, Sawada U, Kura Y, Kodama F (1997). Hepatitis B virus carriers in the treatment of malignant lymphoma: an epidemiological study in Japan. Ann Oncol.

